# Strength Degradation in Curved Fiber-reinforced Polymer (FRP) Bars Used as Concrete Reinforcement

**DOI:** 10.3390/polym12081653

**Published:** 2020-07-24

**Authors:** Thanongsak Imjai, Reyes Garcia, Maurizio Guadagnini, Kypros Pilakoutas

**Affiliations:** 1 School of Engineering and Technology, and Center of Excellence in Sustainable Disaster Management, Walailak University, Nakhonsithammarat 80161, Thailand; 2 School of Engineering, The University of Warwick, Coventry CV4 7AL, UK; reyes.garcia@warwick.ac.uk; 3 Department of Civil and Structural Engineering, The University of Sheffield, Sheffield S1 3JD, UK; m.guadagnini@sheffield.ac.uk (M.G.); k.pilakoutas@sheffield.ac.uk (K.P.)

**Keywords:** Curved FRP bars, bent fiber-reinforced polymer (FRP), bend capacity, bend strength, bent test, strength and testing of materials, material characterization

## Abstract

Steel reinforcements in concrete tend to corrode and this process can lead to structural damage. Fiber-reinforced polymer (FRP) reinforcements represent a viable alternative for structures exposed to aggressive environments and have many possible applications where superior corrosion resistance properties are required. The use of FRP rebars as internal reinforcements for concrete, however, is limited to specific structural elements and does not yet extend to the whole structure. The reason for this relates to the limited availability of curved or shaped reinforcing FRP elements on the market, as well as their reduced structural performance. This article presents a state-of-the art review on the strength degradation of curved FRP composites, and also assesses the performance of existing predictive models for the bend capacity of FRP reinforcements. Previous research has shown that the mechanical performance of bent portions of FRP bars significantly reduces under a multiaxial combination of stresses. Indeed, the tensile strength of bent FRP bars can be as low as 25% of the maximum tensile strength developed in a straight counterpart. In a significant number of cases, the current design recommendations for concrete structures reinforced with FRP were found to overestimate the bend capacity of FRP bars. A more accurate and practical predictive model based on the Tsai–Hill failure criteria is also discussed. This review article also identifies potential challenges and future directions of research for exploring the use of curved/shaped FRP composites in civil engineering applications.

## 1. Introduction

Since the late 1980s, fiber-reinforced polymer (FRP) reinforcements have emerged as an alternative to replace conventional steel bars in reinforced concrete (RC) structures [1,2,3,4,5,6,7,8]. Since FRP reinforcements do not corrode and are very durable, they can extend the structures’ service life and reduce the maintenance/repair costs of concrete structures [9,10,11,12,13,14,15]. To date, internal FRP reinforcements for concrete are mainly limited to specific structural applications, such as bridge decks, road barriers, marine structures, and tunnel and underground infrastructure. The limited use of internal FRP reinforcements could be partly due to the lack of commercially available curved or shaped reinforcing elements needed for complex structural connections [16,17], concerns with durability issues [18,19,20,21,22,23], and the potential degradation of fiber/matrix compositions when FRP reinforcements are exposed to fire [20,21,24,25,26,27,28,29].

**Figure 1:**
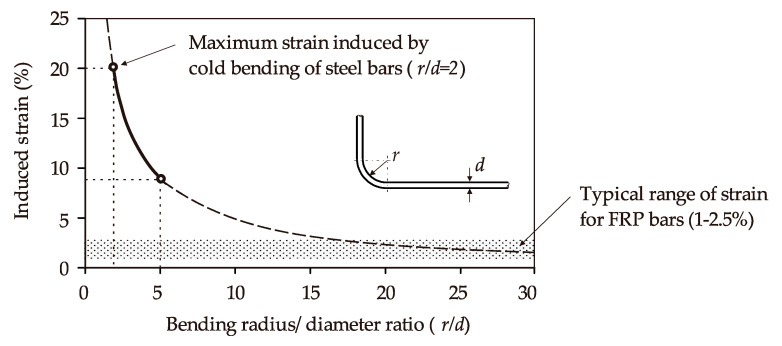
Induced strain values in cold-bent bars (adapted from Imjai et al. [38]).

In current construction practice, most curved/shaped steel bars are pre-bent and pre-cut to the right shapes and lengths off-site. Unlike FRP reinforcements, steel bars have an elastoplastic behavior and can thus be easily shaped by cold bending. Existing guidelines for the cold bending of steel bars (e.g., BS 8666 [30]) specify a bend radius to diameter ratio (*r/d*) of 2 for mild steel, which can induce a maximum strain value of 20% in the steel (see Figure 1). In the case of cold-bent FRP reinforcements, however, there are problems associated with the potential buckling of fibers located on the compression side.

Moreover, the typical ultimate strain value of commercial FRP composites used as embedded reinforcements in concrete structures varies from 1% to 2.5%. Hence, the induced strain in the fibers needs to be controlled to avoid premature failure of the reinforcement [31,32,33]. As a result, cold bending of FRP reinforcements requires larger *r/d* ratios than those currently specified for steel reinforcements [16,34,35,36,37].

**Figure 2:**
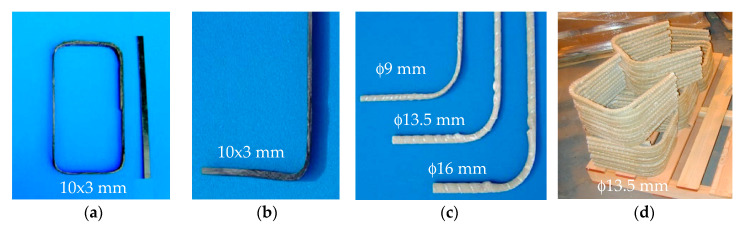
Commercially available curved fiber-reinforced polymer (FRP) reinforcements: (**a**) thermoplastic FRP stirrup; (**b**) J-hook FRP strip; (**c**) pre-bent FRP bar; and (**d**) U-shaped FRP bar.

To date, only a few commercially available FRP bars are supplied in bent configurations, and all of them are pre-bent during manufacturing. Bends are usually created while the material is partially cured. Typical bent shapes available include thermoplastic FRP stirrups [38] (Figure 2a), J-hook thermoplastic FRP strips, pre-bent GFRP thermoset composites (Figure 2b,c), and U-shaped thermosetting FRP bars [16,38,39] (Figure 2d). Whilst carbon (CFRP), glass (GFRP), aramid (AFRP), and basalt (BFRP) bars exist on the market, CFRP and GFRP seem to be much more widely used in actual RC applications and research [40]. This is understandable since CFRP has better properties than all of the other composites, whereas GFRP is significantly cheaper than other composites [8,36,41,42].

Whilst FRP materials work most effectively when subjected to pure axial tension, most FRP RC structures are subjected to a combination of stresses. Previous studies have reported that the tensile strength of FRP reinforcements reduces under a combination of tensile and shear stresses [32,33,43,44,45,46,47,48,49,50,51,52,53]. This becomes an issue in curved FRP reinforcements in RC structures, since premature failures can occur at the bent corner, as reported in the existing literature [33,43,53,54,55,56]. Indeed, the results from such research studies have shown that the tensile strength of a bent portion of an FRP bar can be as low as 25% of the maximum tensile strength of that developed in the straight part. Strength degradation that occurs at the bent portion of an FRP bar can be quantified using empirical equations, such as the one initially proposed by the Japanese Society of Civil Engineering (JSCE) [45], which is currently adopted in other design guidelines. To account for this potential failure, several design guidelines ([34,36,45,57,58]) limit the design strain values in the case of curved FRP reinforcements in RC structures. However, equations included in the current design guidelines to predict strength degradation at the bent portion of an FRP bar were empirically derived and are mainly a function of the bend geometry. The results given by such equations do not seem to yield consistent results when different types of composite are used [59]. As a result, there is a need to reassess the accuracy of such equations in light of the existing and new experimental evidence.

This article provides an overview of existing and ongoing research on the strength of curved FRP reinforcements in RC structures. Extensive experimental works investigating the strength degradation of curved FRP composites are chronologically presented. Test data available from the literature are also included in the appendix as an additional source. Modern techniques used to fabricate customized/complex shaped FRP composites are also discussed as emerging challenges.

## 2. Research on the Strength Degradation of Curved FRPs

**Figure 3:**
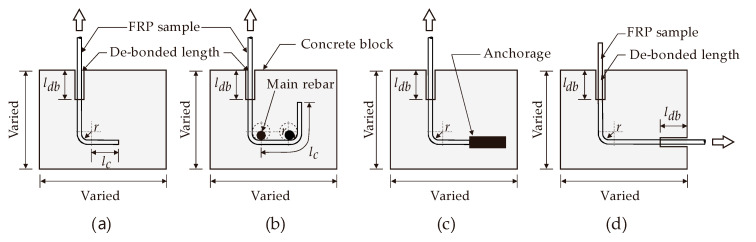
Different pullout setups for examining the bend capacity of FRP reinforcements; (**a**) J-hook specimen, (**b**) U-shaped specimen, (**c**) J-hook specimen with anchorage, and (**d**) J-hook specimen with unbonded unloaded end (illustration adopted from [38]).

Pioneering research by Ozawa et al. [60] examined the “*bend capacity*” of curved FRP reinforcements by testing concrete beams. The concrete beams were reinforced with flexural and shear FRP reinforcements. The reinforcements consisted of continuous glass and carbon fibers impregnated with resin and formed by filament winding. A total of 10 beam specimens were tested under two-point bending; two of them were statically loaded and the other eight were fatigue loaded. The authors reported that, if the beams failed in shear, FRP stirrups could fail at the bent portion at a stress lower than the ultimate strength of the equivalent straight bar. Ozawa et al. concluded that the stress concentration that developed at the bent portion of the bar caused rupture—a failure which originated from the inside of the bend. Similar conclusions were also reported in a subsequent study by Miyata et al. [61], after carrying out a series of pull-out tests that studied the effect of bends on hybrid FRP bars embedded in concrete blocks (see Figure 3a). Direct tensile tests were performed on the reinforcement, which consisted of a 10 mm-diameter hybrid FRP composite made of continuous glass and high-strength carbon fibers impregnated with resin. The main parameter investigated was the variation of the tensile strength of bent FRP bars as a function of the internal bending radius (*r*). Five different bar diameters were used in the test and the bending radius was set to three times the bar diameter (i.e., *r/d = 3*). The authors reported that most of the bent specimens failed due to the rupture of the FRP bars at the bent section, and that the fibers started to break from the inside portion of the bend. They also concluded that the failure load increased as the internal bending radius increased. Although the studies by Ozawa et al. [60] and Miyata et al. [61] provided some insight into the strength degradation of bent FRP bars, the tests only considered a few test parameters and their conclusions were therefore not general. Additionally, these tests did not consider the bond contribution along the bent portion and the effect of tail anchorage. Other parameters that could affect the bond stress, such as the concrete strength and surface treatment of FRP bars, were also excluded in these tests.

To examine the factors that influence the shear capacity of concrete beams with FRP stirrups, Nagasaka et al. [47] tested 35 half-scale beams internally reinforced with FRP bars. The parameters investigated were the type and reinforcement ratio of FRP stirrups, as well as the concrete strength. Nagasaka et al. also tested four panel specimens to investigate the bend capacity of FRP stirrups with the main reinforcement, so as to simulate the bond at the bent location around the main bar (see the pullout arrangement shown in Figure 3b). The FRP bars were aramid, carbon, glass, and hybrids of glass and carbon FRP. The vertical leg was left unbonded to the beginning of the bent portion, and the bend radius was two times the bar diameter (r/d = 2). Nagasaka et al. reported that the ultimate shear capacity of concrete beams reinforced with FRP stirrups was determined by the tensile rupture of stirrups at the curved sections, or by crushing of a concrete strut formed between diagonal cracks. They also found that the tensile strength of curved FRP bars was only 25%-80% of that of a straight counterpart. One of the main contributions of Nagasaka et al.’s study is the finding that the degree of bend capacity reduction depends on the material compositions of the FRP composite.

Similar tests were carried out by Maruyama et al. [43], who tested 14 bent FRP samples embedded in concrete blocks with a 50 mm embedment length (*l_db_*) and an anchor at the end of the tail to improve bonding (Figure 3c). The main parameters studied were different types of composite materials, bending radii, and the concrete strength. Curved pultruded CFRP rods, seven-strand CFRP rods, and braided AFRP rods were tested in direct tension and compared to steel bars with similar configurations. The bending radii (*r*) considered in this study were 5, 15, and 25 mm for each type of rod. Two different concrete strengths were used (*f’_c_ =* 50 and 100 MPa) for each type of FRP rod. It was reported that all of the specimens failed due to rupture of the composite at the start of the bend on the loading side. All of the bend capacities of FRP bars were 48–82% lower than the tensile strengths of the straight portions. Moreover, the bend capacity trended to decrease hyperbolically as the bending radius decreased, and the bend capacity of FRP specimens increased in higher strength concrete and became more pronounced with seven-strand CFRP rods and braided AFRP rods. This may have been due to the better bond developed by the stranded and braided composites, and the resulting lower amount of tensile stress transferred to the bend. In the case of pultruded CFRP rods, the concrete strength had little effect on the bend capacity. This may have been because the bond given by the roving wrapped around the rod was lost during the pullout tests, and adhesion at the bar-concrete interface thus became less significant. The authors also reported that the tensile strength at the bend varied with the type of fiber and the method of bending. The highest bend capacity-to-strength ratio was mobilized by braided AFRP rods, followed by strand CFRP and pultruded CFRP rods. These results indicated that the bend capacity depended on the type of FRP and the reinforcement surface (i.e., on bond properties). It should be mentioned that the test results of Maruyama et al. [43] were later used to calibrate the predictive equation for calculating the bend capacity of FRP reinforcements in JSCE’s guidelines [45]. Such an equation is also included in the current ACI guidelines [62] to predict the bend strength of FRP bars.

Ehsani et al. [46] investigated the bond behavior of 90^o^ degree-hooked GFRP bars in concrete through thirty-six direct pullout tests such as those shown in Figure 3d. The main parameter examined in Ehsani et al.’s study was the relationship between the strength capacity of curved FRP bars and the concrete compressive strength (*f’_c_*), which varied between 28 and 56 MPa. Other examined parameters included the bend radius to FRP bar diameter ratio (*r/d* = 0,3) (diameters, *d =* 9.5, 19.0, and 28.6 mm), embedment length, and tail length (*l_c_*) beyond the hook. In these tests, the tensile load was horizontally applied through a gripping system (Figure 3d). Ehsani et al. found that the bend capacity was highly affected by the bend radius and bar diameter. When using *r/d* = 3, the bend capacity ranged from 64% to 70% of the ultimate tensile strength and the bend capacity tended to increase when a higher concrete strength was used. Based on their results, the authors recommended a minimum bend radius of *3d* for GFRP hooks, as well as a tail length of *12d*, since the tail length beyond *12d* had no beneficial effect on the strength of the bent bar. As the bend capacity increased with the embedment length, Ehsani et al. also recommended a minimum development length of *16d* for a 90^o^ standard GFRP hook. The results from this study confirmed that the concrete strength, embedment length, and tail length are important parameters that influence the bent portion of FRP bars. Unfortunately, the study by Ehsani et al. [46] did not consider the types of composite used or the different bending geometries that could affect the bend capacity of FRP bars.

The effectiveness of a bent FRP reinforcement depends on the bond characteristics of the reinforcement itself, but also on the characteristics of the embedment and tail lengths. Accordingly, Vint and Sheikh [33] examined the bond performance of GFRP bars with different anchorage configurations (90° degree-hooked bars and straight bars with mechanical anchor heads). A total of 72 pullout GFRP specimens (as shown in Figure 3b) were tested using different anchorage configurations: Straight anchorage, mechanical anchor heads, or bends. Bent GFRP bars with different bending radii and surface coatings were used to examine the performance of this anchorage solution. Vint and Sheikh concluded that a full tensile strength in the fiber direction could be developed for bonded lengths of 5*d* in specimens with bent bars and 10*d* for specimens with an anchorage head. However, the bend capacity of the GFRP bars was only 58–80% of the ultimate tensile strength of the straight portion. This indicates that, although mechanical anchor heads could potentially enhance the bond behavior of bent FRP bars, the theoretical ultimate tensile strength of the bars cannot be achieved. 

The above mentioned studies examined the bend capacity of FRP bars using geometries typical of end anchorages (e.g., a relatively large corner radius). However none of the previous studies tested FRP reinforcements with geometries similar to those used in steel stirrups [16,33,38].

Previous research has also studied the effect of bends in FRP stirrups, but using geometries similar to those used in conventional steel stirrups [48,51,59,63,64,65,66,67,68,69,70,71,72]. In these conditions, the tight corner radius of FRP stirrups tended to limit the shear capacity of the concrete beams, where premature failure was generally observed in the proximity of the bent portion [51,64,72]. To study the failure behavior of thermoplastic FRPs as shear reinforcements in concrete beams, Currier et al. [73] carried out bent tests on thermoplastic FRP stirrups made of nylon/carbon and nylon/aramid FRP fibers formed using a thermoplastic matrix resin during the pultrusion process. The thermoplastic FRP strips were bent in the laboratory by applying heat to create the closed shape of shear links, having an internal bending radius of 12.7 mm. The bend capacity of the thermoplastic FRP links was evaluated using a test setup similar to the ACI B.5 method. The bend capacity of the thermoplastic FRP bars was 25% of the ultimate tensile strength of the straight portion, and failures on all of the tested specimens were observed at the bent portion of the stirrup.

Ueda et al. [65] investigated the performance of FRP stirrups partially embedded into a concrete block, which aimed to simulate a shear crack crossing the FRP stirrups. The 6 mm-diameter FRP rods used in Ueda et al.’s study were braided, epoxy-impregnated aramid fibers. The main variables in the study were the embedment length and the distance from the artificial crack to the bend. Tensile forces were transferred through steel plates and by steel rods to the bearing plates. The test setup was adopted from the ACI B.5 method, except that the free distance between two concrete blocks was not 200 mm, but the artificial crack width instead. The artificial crack initiated with a 0.5 mm gap and began to open as the tensile forces were applied in the bent portions of the FRP sample. Ueda et al. also conducted Finite Element Analyses (FEA) to assess the nature of the stress–strain fields developed in the bent region. Their results showed that the bend capacity varied between 40% and 100% of the ultimate capacity in the direction of the fibers. The FEA showed that high strains developed in the inner portion of the bend, which was assumed to be the location of failure initiation. For an embedment length of 100 mm, the failure stress was higher than the nominal strength of the straight bar. The numerical analysis performed by Ueda et al. was perhaps the first that focused on the stress–strain field at the bent portion of FRP bars. Their results also agreed with previous research where premature failure mostly initiated at the proximity of the bends.

Morphy et al. [53] tested sixteen specially-designed specimens using different types of FRP stirrups by employing the ACI B.5 method [74]. The parameters investigated were the type of FRP material, bar diameter, stirrup anchorage and embedment length of the stirrup in the concrete, and the configuration of the stirrup anchorage. Three types of FRP reinforcements were used: Carbon FRP Leadline bars, Carbon Fiber Composite Cables (CFCC), and GFRP bars (C-BAR). All of the bent stirrups were embedded in concrete blocks with *f’_c_ =* 45 *MPa*. The embedment length within the block varied by the debonding part of the stirrups. The authors found that a decrease in the embedment length increased the tendency of failure at the bent region of the stirrup, which resulted in a bend capacity of 40% of that developed in a straight bar. From the results, it was suggested that a 150 mm embedment length was sufficient to achieve the full strength in the direction of the fibers. Morphy et al. also found that when a large bending radius to bar diameter ratio (*r/d*) is used, a higher bend capacity is observed. Based on their test results, and using the stirrup spacing recommended by the ACI codes [75], they proposed to limit the strength of CFRP stirrups to 50% of the unidirectional tensile strength, in order to account for strength degradation due to the bend.

**Figure 4:**
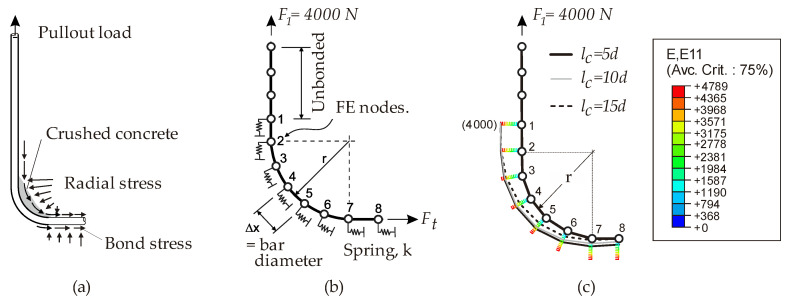
Physical model vs. mathematical finite element (FE) model for a bent FRP bar; (**a**) pullout test on J-hook FRP bar, (**b**) modelling of bond along FRP/concrete interface, and (**c**) strain distribution along the bent portion of FRP bar.

More recently, Imjai et al. [33] studied the bend capacity on bent FRP stirrups using the pullout test shown in Figure 4a. A total of 47 bent thermoset and thermoplastic FRP bars with 19 different configurations were investigated. The parameters investigated included the ratio *r/d*, surface treatment, embedment length (*l_b_*), and concrete strength (*f’_c_*). It was found that the capacity of the curved FRP composites could be as low as 25% of the ultimate tensile strength of the material parallel to the fibers. Based on the results, it was recommended that a minimum ratio *r/d =* 4 was used to guarantee that the composite could resist 40% of its unidirectional tensile strength parallel to the fibers. Imjai et al. also conducted FEA to study the bond stress along the bent portion of an FRP bar embedded in concrete. The bond mechanism between the bent bar and the concrete was explicitly modeled with identical non-linear spring elements, with the stiffness determined from the load-slip characteristic obtained from the pullout tests (Figure 4b). The FEA results confirmed that high stress concentrations develop at the start of the bent portion, thus indicating that failure could be expected to occur at this location (Figure 4c). However, by using a larger bending radius or providing a sufficient bond along the bent portion, the stress concentration at the start of the bend can be significantly reduced, and a higher bend capacity can be achieved.

Although all of the studies summarized above examined the behavior of curved FRP bars embedded in concrete structures such as stirrups or anchorages, whereas externally bonded FRP reinforcements (EBR) are widely used as strengthening material in RC structures [76,77,78]. In this situation, the EBR provides additional confinement and/or shear capacity around members, and thus may also suffer from the bent effect at the member corners. The need to bend the composites may deteriorate the performance of the FRP laminate and the efficiency of its confining/strengthening action. Yang et al. [79] studied the effects of the corner radius on the strength of FRP lamina using a test setup similar to the ACI B.12 [74]. In their experimental program, one and two plies CFRP lamina were applied by the manual lay-up procedure over interchangeable corner inserts. They concluded that the corner radius (*r*) affects the strength of CFRP laminates. The test results showed that only 67% of the ultimate laminate strength could be developed when a large-radius insert was used. As the corner radius was decreased, the strength capacity of the FRP lamina further reduced. A higher failure stress was achieved by increasing the number of layers used.

Most research studies available in the literature have investigated the performance of FRP reinforcements at ambient room temperature [80,81,82,83]. However, the low glass transition temperature of FRPs makes them very susceptible to high temperatures. Fire exposure can lead to a rapid loss of mechanical properties, such as the stiffness and strength [84,85,86]. To study the mechanical properties of FRPs exposed to fire, Abbasi and Hogg [24] tested two full-scale RC beams (350 × 400 mm with a span of 4400 mm) reinforced with both thermoset and thermoplastic GFRP reinforcements in flexure and shear. The beam specimens were heated on three sides using a maximum temperature of 462 °C. The furnace temperatures were recorded, monitored, and controlled to follow the standard fire curve in accordance with BS 476: Part 20 [87]. It was recommended that a minimum clear concrete cover of 70 mm is required to meet the fire design requirements for the minimum periods of fire resistance (fire endurance) of up to 90 min. Using the experimental result from Abbasi and Hogg [24], Hawileh and Naser [88] developed a 3D nonlinear finite element (FE) model built from their previous studies [89,90] to predict temperature and mid-span deflection of a GFRP RC beam when exposed to fire. From their transient thermal-stress finite element analysis, it was recommended that the FE model was used to predict the mechanical performance of FRP RC beams when exposed to fire when fire endurance is required [91].

Based on the literature summarized in this section, it is evident that relatively little information is available to develop accurate predictive models for curved FRP reinforcing bars [60,61,92]. Whilst different test configurations were used in examining the bend capacity of FRP bars, the majority of studies used pullout tests on bent FRP bars embedded in concrete specimens, such as those shown in Figure 3. It is also evident that numerous factors affect the bend capacity of FRP reinforcements, such as the bend geometry, materials from which the type of composite is made, concrete strength, and bond stress between the concrete/FRP bar interface. Advanced FE techniques were used to study the stress–strain field along the bent portion of FRP bars and the results confirmed that premature failure always initiated at the proximity of the bends, which confirmed the reports from companion works in the literature. However, issues such as mechanics at a macro-scale of the material composition of the composite bent portion when subjected to external loads, irregular shape, and cross-section and bond stress along the bent portion have not yet been investigated and are a matter of future research. The results from the tests discussed in this section have also been reflected in the development of a predictive equation included in the current design guidelines, as discussed in the following section.

## 3. ACI Testing Methods to Determine the Bend Capacity of FRP Reinforcements

**Figure 5:**
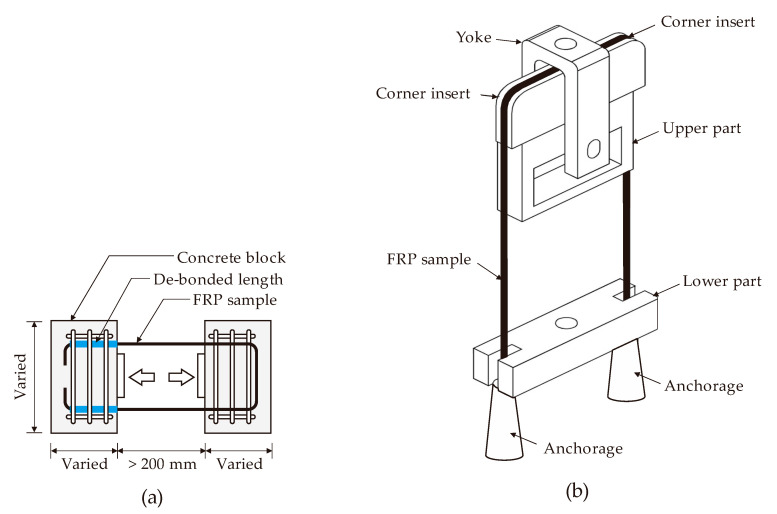
ACI test method: (**a**) B.5 and (**b**) B.12.

Different tests have been proposed to calculate the strength reduction in bent bars. For instance, ACI 440.3R [74] proposes using the B.5 method (bent bar capacity) and the B.12 method (corner radius), as illustrated in Figure 5a,b, respectively. The B.5 method measures the ultimate capacity of the FRP by testing (in tension) the straight portion of an FRP C-shaped stirrup whose bent ends are embedded in two concrete blocks (Figure 5a). The bend capacity of bent FRP bars is measured and compared to the ultimate tensile strength of the bar to obtain the strength reduction factor due to bend effects. The B.12 method measures the effect of the corner radius on the tensile strength of the FRP bar using the testing apparatus shown schematically in Figure 5b. The apparatus applies tension in the U-shaped FRP that reacts against the bent portion mounted on a yoke.

ISIS Canada [93] and ACI 440.6M-08 [94] suggest using either of the ACI B.5 or B.12 methods to determine the bend capacity of curved FRP reinforcements. Ahmed et al. [51] compared the two ACI methods by testing four CFRP stirrup specimens using the B.5 method and 12 GFRP stirrup specimens using the B.12 method. Ahmed et al. concluded that the B.5 test method led to more realistic results for the bend capacity of the FRP stirrups because the test arrangement better simulates the actual mechanism of stirrups embedded in concrete. The ACI B.12 method led to more realistic results when the FRP composite was applied externally.

Based on the review in this section, it is evident that the ACI guidelines only provide two standard test configurations for assessing the bend capacity of FRP reinforcements. Out of these two, the ACI B.5 method is the most feasible for examining the bend strength of FRP composites used as shear reinforcements. In reality, however, simple pullout tests have been widely used in the literature due to the fact that the setup can be practically achieved and parameters such as the bend geometry, embedment length, and tail length can be easily installed in the setup. Conversely, the ACI B.5 and B12 methods require more detailing in the test setup and the eccentricity of the applied loads has to be carefully monitored. The results of the bend tests performed by several test methods in the literature were used in the process of the development of the predictive model for the bend capacity and will be described in detail in the following section.

## 4. Models to Assess the Bend Capacity of FRP Reinforcements

In 1995, Nakamura and Higai [52] conducted a theoretical study on the bend capacity of FRP stirrups based on test results from Miyata et al. [61]. As a result of their study, the authors proposed an empirical model to calculate the bend capacity of FRP composites (*f_b_*), as shown in Table 1; Equation (1). The model primarily depends on the bend ratio r/d, and therefore neglects the variation of the composite cross-section, the type of composites, and the influence of bond characteristics between the FRP/concrete interface.

Based on test results from Ueda et al. [65], Ishihara et al. [50] analysed the behavior of bent FRP stirrups embedded in concrete using a 2D FEA. The results of their study showed that the strength of a bar at its bent portion directly increases with the radius of the bend. Based on an FEA parametric study, Equation (2a,b) was proposed to assess the strength of the bent portion (*f_b_*). Note that Equation (2b) is a special case of Equation (1) in which *λ* replaces *d/r*. The study by Ishihara et al. showed that the reduction in bend strength was also a function of the different types of FRP composites. Ishihara et al. suggested that bond characteristics and differential slippage of the FRP rod (which were not considered in their FEA) could play an important role in strength reduction. 

**Figure 6:**
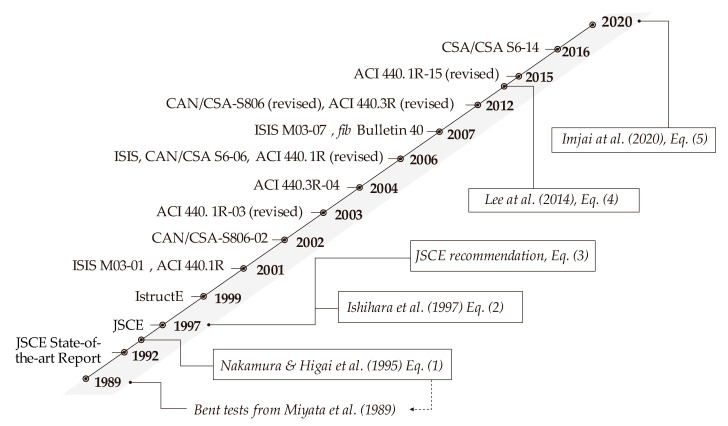
Chronological development of a predictive model for bend capacity and code provisions.

The chronological development of predictive models for the bend capacity and evolution of design guidelines is shown in Figure 6. The initial JSCE guidelines were based on early work by Japanese researchers. In North America, the ACI and ISIS recommendations were mainly influenced by the work of American-based researchers. It is also evident that the development of research accompanied the development of design guidelines, but only until the early 2010′s. Accordingly, none of the current guidelines reflect the state of the art in the subject.

**Figure 7:**
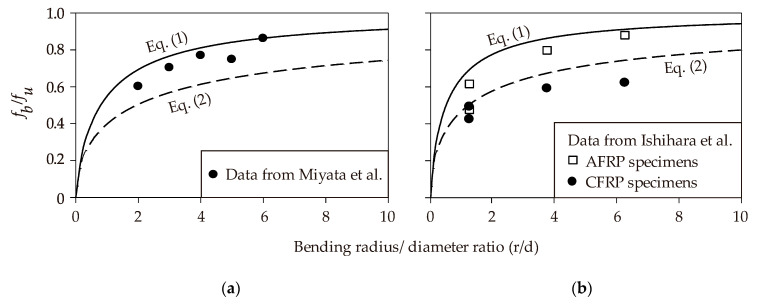
Predicted bend capacity of FRP bars from Nakamura and Higai’s (**a**) and Ishihara et al.’s (**b**) models.

Figure 7a,b compare, respectively, the predictions given by Equations (1) and (2a) and test data from Miyata et al. [61] and Ishihara et al. [50]. The results show that the experimentally-derived bend capacity increases with an increasing *r/d* ratio. Figure 7a also shows that the predictions from Equation (1) agree better with the test results when compared to Equation (2a). This is not surprising as Equation (1) was empirically derived using test data from Miyata et al. [61]. In Figure 7b, it can be observed that Equation (2a), as proposed by Ishihara et al. [50], predicts the experimental results more accurately than Equation (1). This is because the equation proposed by Ishihara et al. [50] was empirically derived using their own test data.

**Figure 8:**
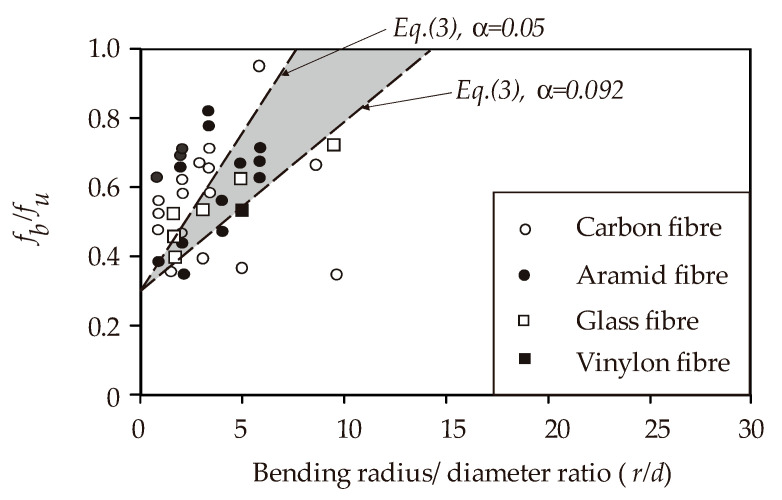
Bend capacity by the Japanese Society of Civil Engineering’s (JSCE’s) equation (data from JSCE document [45]).

The strength degradation at the bent portion of FRP composites is often quantified using Equation (3) (see Table 1), which is included in current design recommendations for concrete structures reinforced with FRP composite materials [34,36,94,95,96]. It should be noted that Equation (3) is based on the JSCE guidelines [45]. In Equation (3), the strength of the bent portion, *f_b_*, is solely expressed as a function of the uniaxial tensile strength of the composite, *fu*, and the bar geometry (i.e., bar diameter, *d*, and bend radius, *r*). The strength of the bent portion varies greatly, even for the same type of fibers, depending on the bending characteristics and type of resin used. Therefore, the strength of the bent portion should be determined on the basis of suitable tests. The regression line in Figure 8 is supposed to give an adequate margin of safety. It should be noted that Equations (1) to (3) are only applicable to circular FRP bars.

More recently and based on modifications to Equation (3), Lee et al. [48] proposed Equation (4) to calculate the bend capacity of non-circular FRP sections. Non-circular bars are converted into equivalent circular bars using an equivalent diameter with the safety factor (Fs). The safety factor, Fs, is given different values, such as Fs = 1.3 in JSCE [45] and 1.5 in ACI440.1R-15 [62], CAN/CSA S6–06 (CSA 2006), and ISIS-M03-07 [96]. Lee et al. also proposed different values of α (suitable for Equation (3)) using linear regression analysis from 14 tests. The researchers validated their model (Equation (4)) using previous ACI B.5 bent test data from the literature [49,51,64].

It should be noted that Equations (1) to (4) are empirical and only depend on the geometry of the bend, whilst the bond characteristic between the FRP bar/concrete interface, type of FRP, and material composition are neglected. Recent research by Imjai et al. [59] demonstrated that the predictions of Equations (1) to (4) do not match the experimental data available in the existing literature. As a result, Imjai et al. proposed a new macromechanical-based equation (Equation (5a)) that more accurately calculates the bend capacity of a bent FRP reinforcement. Equation (5a) adopts the Tsai–Hill failure criterion [42,97] for a unidirectional orthotropic lamina with fibers in one-direction and subjected to plane stress in the 1–2 plane. The bend capacity (*f_b_*) is expressed as a function of the strength reduction factor (*k*) multiplied by the ultimate strength parallel to the fibers (*f_u_*). The strength reduction factor (*k*) is less than unity and ranges from 0.25 to 0.70, depending on the value of *β* (e.g., Equation (5b)). The factor *β* is the ratio of the longitudinal tensile strength and transverse compressive strength of the FRP material. In their model, the factor *β* is explicitly derived from the Tsai–Hill failure criterion [97], which represents the physical meaning of materials at the macro-scale, and the type of composite/resin composition is considered when determining the bend capacity of unidirectional FRP composites [59,97,98].

Appendix A compares the bend capacity calculated by the different equations in Table 1 against test data available in the literature. In Equation (5), the strength reduction factor *k* used in the calculations depends on the parameter *β,* which is the ratio of the longitudinal tensile strength (*f_u_*) and the transverse compressive strength (*fc_T_*). The values of *fc_T_* were not available for any of the composite specimens summarized in Appendix A. Accordingly, a value *β* = 7.5 is recommended as the back-calculation of the transverse compressive strength in the range 80–246 MPa, which lies within the typical range for FRP composites reported in the literature [59]. 

**Figure 9:**
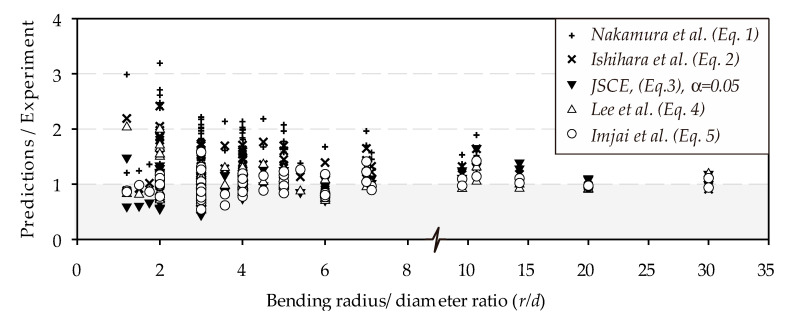
Prediction of experimental tests using existing code provision and models.

Figure 9 compares 80 test results from the literature and results calculated with the equations in Table 1. The comparative results presented in Figure 9 clearly show that the JSCE’s equation (*α* = 0.05) is conservative, with a mean prediction/experiment ratio of *P/E* = 1.02 and a standard deviation of *SD* = 0.27. It can also be seen that the five equations yield quite different ranges of results. For instance, Equations (1) and (2) overestimate the bend capacities for the data in the literature, as shown by *P/E* = 1.66 and *SD* = 0.46 for Equation (1), and *P/E* = 1.34 and *SD* = 0.33 for Equation (2). In comparison, Equation (4) better predicts the test results and has less scatter (*P/E* = 1.08, *SD* = 0.28). Equation (5) shows the best agreement with the test results and has a low scatter (*P/E* = 1.00, *SD* = 0.25). The differences between the calculated values can be attributed to differences in the original formulation of the empirical equations that can be attributed to the influence of the types of composites used in the experimental program.

## 5. Prefabricated FRP Composites and Future Challenges

**Figure 10:**
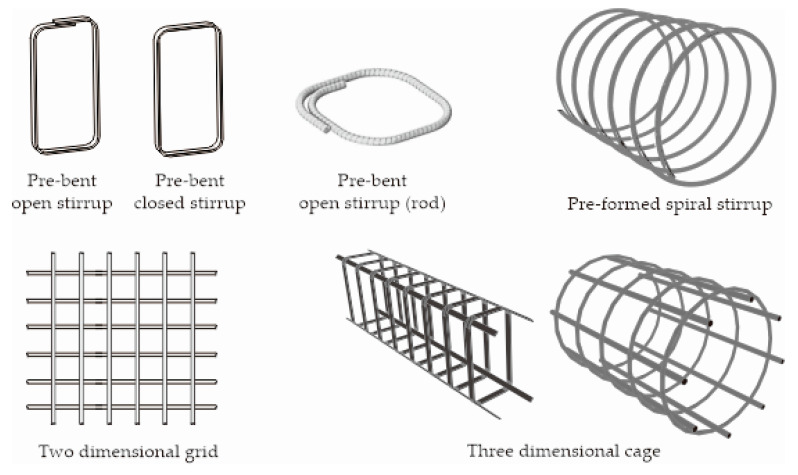
Various commercially available shaped FRP reinforcements (adopted from [38]).

In the past, the methods employed to manufacture complex or customized FRP shapes were very expensive and required a complicated manufacturing process. Nowadays, with the aid of computer automatic control and 3D printing, various shaped FRP reinforcements (shown in Figure 10) are currently available on the construction market. An advanced filament winding manufacturing process has been developed, in which resin-impregnated fibers are wound onto specially-designed mandrels to produce customized closed shapes such as shear stirrups [55,99]. In these pre-bent closed loop stirrups, the material is wound around a mold into one large stirrup. After the completion of the curing process, the mold is removed and the large stirrups are then cut into smaller stirrups of appropriate width links (e.g., pre-bent open/closed stirrup). The advanced filament winding process can produce a tailored FRP reinforcement with a tensile strength exactly where it is needed. Experimental studies by Lee et al. [48,100] have proved that advanced filament winding forms the fibers in wide and thin cross sections suitable for the manufacturing of closed FRP stirrups. This method also allows for the quick and accurate fabrication of reinforcement cages with a consistent quality of material and uniform cross-section. This is because the winding system allows the internal radius of the bend to be tighter than for traditional open stirrups as the fibers do not need to slide over each other, as is required when bending a straight pultruded bar before the resin polymerizes [99,101].

Shear reinforcement is often produced from pultruded bars prior to resin polymerization in circular, rectangular, and other forms, such as a spiral shaped stirrup [102]. A recent study [68] reported that prefabricated 3D FRP reinforcement cages produced using filament winding were successfully used in concrete elements. The manufacturing process of the 3D reinforcement FRP cage included wet and dry winding process. In the wet-winding process, each layer of fiber was impregnated with a two-component epoxy resin, squeezed with a polytetrafluorethylene tool to remove any excess, and wound around the mold. The stirrups were cured at room temperature for 72 h, prior to being demolded. In the dry winding process, the pre-preg tow was wound around the mold, before being packed in a vacuum bag and cured at 120 °C for 4 h. The results obtained from tests on bent reinforcement showed that the use of wound CFRP instead of conventional circular CFRP stirrups offered advantages in terms of construction flexibility at more affordable costs, but it can also help mitigate the strength reduction at bent corners.

Whilst current advanced technology exists to produce complex 3D shaped FRP composites for engineering applications, a gap remains between the feasibility and durability of these engineering products to be used in concrete structures over the design lifetime. Full-scale testing of structural aspects and durability tests should be performed prior to fully exploiting the full functionality of shaped FRP reinforcements in civil engineering applications.

Due to the nature of FRP reinforcement manufacturing and its material anisotropic properties, advanced FEA should also be used to assess the structural behavior of concrete elements when FRP is used as reinforcement.

## 6. Concluding Remarks

This article presents an extensive literature review on the strength degradation of curved FRP composites, and discusses the performance of exiting predictive models for calculating the bend capacity of FRP reinforcements. The literature review indicates that the use of FRP bars as internal reinforcements for concrete is still limited to specific structural elements and does not yet extend to the whole structure. The reasons for this can be related to the limited availability of curved or shaped reinforcing elements on the market and their limited structural performance. Previous studies hwv4 shown that the mechanical performance of bent portions of composite bars significantly reduces under a multiaxial combination of stresses, and that the tensile strength at the bend can be as low as 25% of the maximum tensile strength developed in the straight part. The capacity of the bent specimens does not seem to vary linearly with the *r*/*d* ratio (as currently defined in the JSCE’s equation) and does not appear to only be a function of the bend geometry. Rather, bond characteristics appear to be important in controlling the development of stresses along the embedded portion of the composite and in dictating its ultimate behavior. In a significant number of cases, the equation included in the JSCE guidelines was found to overestimate the bend capacity of FRP bars with Prediction/Experiment (P/E) ratios and Std Dev of up to 1.02 and 0.27, respectively. A more recent practical predictive model based on the Tsai–Hill failure criteria predicted the experimental results more accurately (P/E = 1.0) and with less scatter (Std Dev = 0.25) than the predictions of existing models.

It is worth noting, however, that none of the models considered in this analysis, including the macromechanical failure-based model, account for the influence of the concrete strength, embedment length, and tail length. These parameters are believed to play an important role in determining the behavior of bent bars embedded in concrete and could be responsible for the large variation observed in the test data. Future research should focus on the use of advanced finite element modeling to capture the true behavior of unidirectional FRP composites at the micro level. This includes an input of the full definitions of material properties in both transversal and longitudinal directions. Biaxial tests on FRP composites should be performed in order to obtain the failure surface of the materials. However, the durability of curved FRP reinforcements should be assessed over the design lifetime. An advanced filament winding manufacturing process has been developed, in which resin-impregnated fibers are wound onto specially designed mandrels to produce customized closed shapes and these were successfully used as 3D reinforcement cages for concrete elements. However, the long-term durability should be further investigated before completely replacing internal steel reinforcements in concrete structures.

## Figures and Tables

**Table 1:** Summary of equations used to predict the strength degradation of curved FRP reinforcements.

References		Remarks
Nakamura and Higai [52]		Empirical model derived using test data from Miyata et al. [61].
$f_{b}=\frac{r}{d}\ln\left(1+\frac{d}{r}\right)\cdot f_{u}$	(1)	
Ishihara et al. [50]		Derived using test data from Ishihara et al. [50] and further compared to the numerical results obtained from a 2D FE analysis.
$f_{b}=\frac{1}{\lambda }\ln\left(1+\lambda \right)\cdot f_{u}$	(2a)	
where lnλ=0.90+0.73ln (d/r)	(2b)	
JSCE [45]		Empirical model based on test results obtained by Japanese researchers. Unfortunately, information on these tests is not available for all of the specimens and only selected test data from JSCE extracted from Ishihara et al. [50] are presented in the appendix.
$f_{b}=\left(\alpha \frac{r}{d}+0.3\right)f_{u}$	(3)	
Lee et al. [48]		Equation (4) is a modification of Equation (3), but the former can be applied to non-circular sections. The model uses the diameter of the equivalent circular section by converting non-circular bars to equivalent circular bars, $d_{fi}$. α values were obtained from linear regression analysis from 14 tests.
$f_{b}=\frac{\left[0.02\left(\alpha \frac{r}{d_{fi}}+0.47\right)\cdot f_{u}\right]}{F_{s}}$	(4)	
Imjai et al. [59]		The model adopts the Tsai–Hill failure criterion for a unidirectional orthotropic laminar composite at a macroscopic level and considers force equilibrium at the bent zone. The model is calibrated using test results from 26 tests [33] and subsequently verified against 54 test results available in the literature.
$f_{b}=k\cdot f_{fu}$	(5a)	
where $k=\frac{1}{\sqrt{1+\left(\xi \cdot \frac{1}{r}\right)+{\left(\xi \cdot \frac{1}{r}\right)}^{2}\beta^{2}}}$	(5b)	

$f_{b}$ = bend capacity; $f_{u}$ = ultimate strength parallel to the fibers; *r* = bend radius; α = 0.05 corresponds to a 95% confidence limit; α = 0.092 corresponds to a 50% confidence limit; *d* = nominal diameter of the bars; *d_fi_* = diameter of the equivalent circular section; $F_{s}=$ the safety factor; $\xi =\frac{\pi d}{4}$ or t for circular or rectangular cross-sections, respectively; β = strength ratio; and *k* = strength reduction factor for bent FRP bars.

## Abbreviations

The following abbreviations are used in this paper:

ACI	American Concrete Institute
AFRP	Aramid Fiber-reinforced polymer
BS	British Standard
BSI	British Standard Institute
CEB	Comit Euro-international du Beton
CEN	Comite Europeen de Normalisation
CFCC	Carbon Fiber Composite Cable
CFRP	Carbon Fiber-reinforced polymer
EC	Eurocode
FEA	Finite Element Analysis
FIB	Federation Interationale du Beton
FRP	Fiber-reinforced polymer
GFRP	Glass Fiber-reinforced polymer
ISE	Institution of Structural Engineering
ISIS	Intelligent Sensing for Innovative Structures
JSCE	Japanese Society of Civil Engineers
NFR	Non-Ferrous Reinforcement
OPC	Ordinary Portland Cement
RC	Reinforced Concrete
SLS	Service Limit State
ULS	Ultimate Limit State

## Appendix A

**Table A1:** Bent test data from 1997–2017.

Reference	No.	Type of FRP Specimen	*d* (mm)	*r* (mm)	*d_fi_* (mm)	*r* */d*	*r* */d_fi_*	f_u_ (MPa)	f_b_ (MPa)	Equation (1)	Equation (2)	Equation (3)		Equation (4)	Equation (5)	
												*α* = *0.05*	*α* = *0.092*		*β*set	*β*opt
JSCE [45]	1	Braided AFRP	8	16	8	2.0	2.0	1369	812	1110	840	548	663	698	952	463
	2		6	12	6	2.0	2.0	1142	796	926	700	457	553	582	794	387
	3		8	12	8	1.5	1.5	1369	846	1049	778	513	600	685	830	359
	4		10	12	10	1.2	1.2	1283	775	933	684	462	527	634	683	273
	5		6	12	6	2.0	2.0	1142	824	926	700	457	553	582	794	387
	6	7-stranded CFRP	8	16	8	2.0	2.0	1794	557	1455	1100	718	868	915	596	607
	7		6	12	6	2.0	2.0	1620	552	1314	994	648	784	826	538	548
	8		8	16	8	2.0	2.0	1794	595	1455	1100	718	868	915	596	607
	9		10	12	10	1.2	1.2	2271	553	1652	1211	818	932	1122	474	484
	10		6	12	6	2.0	2.0	1620	485	1314	994	648	784	826	538	548
Shehata et al. [49]	11	7-stranded CFRP	3.59	10.8	3.59	3.0	3.0	1782	916	1538	1201	802	1026	944	1199	838
	12		3.59	10.8	3.59	3.0	3.0	1782	1455	1538	1201	802	1026	944	1199	838
	13		4.4	13.2	4.40	3.0	3.0	1842	983	1590	1241	829	1061	976	1239	866
	14		4.4	13.2	4.40	3.0	3.0	1842	1187	1590	1241	829	1061	976	1239	866
	15		6.22	18.7	6.22	3.0	3.0	1875	1900	1618	1264	844	1080	994	1261	882
	16		6.22	18.7	6.22	3.0	3.0	1875	1421	1618	1264	844	1080	994	1261	882
	17		6.22	18.7	6.22	3.0	3.0	1875	798	1618	1264	844	1080	994	1261	882
	18	CFRP strip	5	15.0	5.00	3.0	3.0	1800	1242	1553	1213	810	1037	954	815	846
	19		5	15.0	5.00	3.0	3.0	1800	715	1553	1213	810	1037	954	815	846
Shehata et al. [49]	20	CFRP strip	5	35.0	5.00	7.0	7.0	1800	1163	1682	1413	1170	1699	1098	1350	1376
	21		5	35.0	5.00	7.0	7.0	1800	988	1682	1413	1170	1699	1098	1350	1376
	22		5	35.0	5.00	7.0	7.0	1800	858	1682	1413	1170	1699	1098	1350	1376
	23	GFRP	12	48.0	12.00	4.0	4.0	713	346	636	509	357	476	392	346	410
El-Sayed et al. [64]	24	CFRP rod	9.5	38.1	9.50	4.0	4.0	1328	701	1186	949	665	888	731	698	764
	25		9.5	38.1	9.50	4.0	4.0	1328	761	1186	949	665	888	731	698	764
	26		9.5	38.1	9.50	4.0	4.0	1328	656	1186	949	665	888	731	698	764
	27		9.5	38.1	9.50	4.0	4.0	1328	596	1186	949	665	888	731	698	764
	28		9.5	38.1	9.50	4.0	4.0	1328	789	1186	949	665	888	731	698	764
	29		12.7	50.8	12.70	4.0	4.0	1224	681	1093	874	612	818	673	643	703
	30		12.7	50.8	12.70	4.0	4.0	1224	539	1093	874	612	818	673	643	703
	31		12.7	50.8	12.70	4.0	4.0	1224	697	1093	874	612	818	673	643	703
Ahmed et al. [51]	32	CFRP rod	9.5	38	9.50	4.0	4.0	1538	712	1373	1099	769	1027	846	712	883
	33	GFRP rod	9.5	38	9.50	4.0	4.0	664	387	593	474	332	444	365	407	381
	34		15.9	63.6	15.90	4.0	4.0	599	404	535	428	300	400	329	367	344
	35		19.1	76.4	19.10	4.0	4.0	533	310	476	381	267	356	293	327	292
Lee et al. [48]	36	CFRP rod	9.5	42.8	9.50	4.5	4.5	1880	778	1698	1373	987	1343	1053	896	1161
	37		9.5	42.8	9.50	4.5	4.5	1880	1014	1698	1373	987	1343	1053	896	1161
Lee et al. [48]	38	CFRP strip	4	14.3	4.51	3.6	3.2	1850	763	1631	1293	886	1163	987	762	987
	39		4	14.3	4.51	3.6	3.2	1850	1012	1631	1293	886	1163	987	762	987
	40		4	28.5	4.51	7.1	6.3	1850	1102	1731	1456	1214	1768	1103	1224	1424
	41		4	28.5	4.51	7.1	6.3	1850	1192	1731	1456	1214	1768	1103	1224	1424
	42		4	42.8	4.51	10.7	9.5	1850	935	1769	1535	1545	1850	1220	1465	1604
	43		4	42.8	4.51	10.7	9.5	1850	1167	1769	1535	1545	1850	1220	1465	1604
	44		3	28.5	3.39	9.5	8.4	1740	1079	1654	1423	1349	1740	1111	1318	1466
	45		3	28.5	3.39	9.5	8.4	1740	1215	1654	1423	1349	1740	1111	1318	1466
	46		3	42.8	3.39	14.3	12.6	1740	1267	1682	1490	1763	1740	1258	1499	1589
	47		3	42.8	3.39	14.3	12.6	1740	1373	1682	1490	1763	1740	1258	1499	1589
	48		0.9	18	1.02	20.0	17.7	1880	1731	1835	1660	1880	1880	1550	1724	1782
	49		0.9	18	1.02	20.0	17.7	1880	1703	1835	1660	1880	1880	1550	1724	1782
	50		0.9	27	1.02	30.0	26.6	1880	1882	1849	1710	1880	1880	1880	1799	1827
	51		0.9	27	1.02	30.0	26.6	1880	1586	1849	1710	1880	1880	1880	1799	1827
Vint and Sheikh [44]	52	GFRP rod	9.43	51	9.43	5.4	5.4	833	555	764	628	475	664	481	701	568
	53		11.93	36	11.93	3.0	3.0	655	522	565	441	295	377	347	450	308
	54		13	23	13	1.8	1.8	912	531	721	540	353	420	461	457	275
Imjai et al. [33]	55	GFRP strip	3	6	3.39	2.0	1.8	720	236	584	442	288	348	364	227	244
	56		3	9	3.39	3.0	2.7	720	309	621	485	324	415	377	318	339
	57		3	12	3.39	4.0	3.5	720	324	643	514	360	481	389	393	414
	58		3	15	3.39	5.0	4.4	720	370	656	536	396	547	402	451	472
	59		3	9	3.39	3.0	2.7	720	316	621	485	324	415	377	318	339
	60		3	15	3.39	5.0	4.4	720	415	656	536	396	547	402	451	472
	61		3	9	3.39	3.0	2.7	720	340	621	485	324	415	377	318	339
	62		3	15	3.39	5.0	4.4	720	399	656	536	396	547	402	451	472
	63		3	9	3.39	3.0	2.7	720	367	621	485	324	415	377	318	339
	64		3	15	3.39	5.0	4.4	720	464	656	536	396	547	402	451	472
	65		3	9	3.39	3.0	2.7	720	299	621	485	324	415	377	318	339
	66		3	15	3.39	5.0	4.4	720	334	656	536	396	547	402	451	472
	67		3	9	3.39	3.0	2.7	720	324	621	485	324	415	377	318	339
	68		3	9	3.39	3.0	2.7	720	345	621	485	324	415	377	318	339
	69		3	6	3.39	2.0	1.8	720	183	584	442	288	348	364	227	244
	70		3	9	3.39	3.0	2.7	720	280	621	485	324	415	377	318	339
	71		3	12	3.39	4.0	3.5	720	301	643	514	360	481	389	393	414
	72		3	15	3.39	5.0	4.4	720	316	656	536	396	547	402	451	472
	73		3	9	3.39	3.0	2.7	720	281	621	485	324	415	377	318	339
Imjai et al. [33]	74	GFRP rod	9	54	9	6.0	6.0	760	611	703	583	456	648	448	494	545
	75		9	54	9	6.0	6.0	760	645	703	583	456	648	448	494	545
	76		9	54	9	6.0	6.0	760	592	703	583	456	648	448	494	545
	77		9	54	9	6.0	6.0	760	617	703	583	456	648	448	494	545
	78		13.5	54	13.5	4.0	4.0	590	382	527	422	295	394	325	296	339
	79		13.5	54	13.5	4.0	4.0	590	345	527	422	295	394	325	296	339
	80		9	54	9	6.0	6.0	760	419	703	583	456	648	448	494	545
Mean value (Prediction/Experiment)										*1.66*	*1.34*	*1.02*	*1.28*	*1.08*	*0.98*	*1.00*
Standard deviation (Prediction/Experiment)										*0.46*	*0.33*	*0.27*	*0.32*	*0.28*	*0.18*	*0.25*

Note: *r* is the internal bending radius, *d* is the nominal diameter (diameter for the circular section and thickness for the strip), *d_fi_* is the transformed diameter, *f_b_* is the experimental average failure stress, and *f_u_* is the ultimate strength of the FRP bar.
